# Clinical Significance of Tumor Microenvironment in Acral Melanoma: A Large Single-Institution Study of Caucasians

**DOI:** 10.3390/jcm10071452

**Published:** 2021-04-01

**Authors:** Aneta Maria Borkowska, Anna Szumera-Ciećkiewicz, Maria Chraszczewska, Kamil Sokół, Tomasz Goryń, Piotr Łukasz Rutkowski

**Affiliations:** 1 Department of Soft Tissue/Bone Sarcoma and Melanoma, Maria Sklodowska-Curie National Research Institute of Oncology, 02-781 Warsaw, Poland; aneta.borkowska@coi.pl (A.M.B.); tomasz.goryn@pib-nio.pl (T.G.); 2 Department of Pathology and Laboratory Medicine, Maria Sklodowska-Curie National Research Institute of Oncology, 02-781 Warsaw, Poland; szumann@gmail.com (A.S.-C.); chraszczewska@gmail.com (M.C.); 3 Diagnostic Hematology Department, Institute of Hematology and Transfusion Medicine, 02-776 Warsaw, Poland; sokol.kamil.89@gmail.com

**Keywords:** acral melanoma, hand or foot melanoma, melanoma, microenvironment, TILs, FOXP3, CD4, C8, PD1, PD-L1

## Abstract

Background: The presence of tumor-infiltrating lymphocytes (TILs) in many studies is associated with a better prognosis in melanoma patients. Programmed death-ligand 1 (PD-L1) expression has a significant value in predicting several cancers, but its role in melanoma remains ambiguous. The study aims to report a comprehensive analysis of TILs characteristics and their impact on survival in primary acral melanoma (AM). Methods: Clinical and pathological features and survival outcomes were investigated in 70 patients with AM. Immunohistochemical quantitative analysis of TILs, including expression of CD4, CD8, FOXP3, PD-1, and PD-L1, on melanoma cells was performed. Results: Kaplan-Meier analysis showed significant differences in overall survival (OS) for CD4+ (*p* = 0.021), CD8+ (*p* = 0.037), FOXP3+ (*p* = 0.007), and TILs density (*p* = 0.043). In univariate analysis of immunohistochemical features, FOXP3, CD4, CD8, PD-1, and Melanoma Institute of Australia (MIA) grading TILs (grade, density, and distribution) were correlated with survival. The higher density of FOXP3-positive cells was an independent factor associated with better survival. Conclusions: High TILs content (classed as brisk Clark scale and marked/diffuse TILs MIA grade) regardless of its immunophenotype was associated with better survival outcomes in AM. PD-L1 expression on tumor cells did not influence OS and was independent of clinical and pathological characteristics. We demonstrated that TILs are significant biomarkers in sentinel lymph node status prediction.

## 1. Introduction

Melanoma, with increasing incidence rates, is the most aggressive skin neoplasm and causes most skin cancer-related deaths [1,2]. Cutaneous melanoma located on the acral part of extremities—acral melanoma (AM)—represents a rare subgroup in Caucasians, representing less than 10% of all site melanomas [3]. AM seems to have a worse prognosis than melanomas of other sites, with five-year overall survival (OS) in a range of 59–63% [4,5]. Although the American Joint Committee on Cancer (AJCC) is a globally used system for clinical staging, other factors and biomarkers that could refine prediction are still under research [6].

Melanoma is considered one of the most immunogenic tumors because of its ability to stimulate complex immune reactions. The melanoma tumor microenvironment (TME) comprises various immune cells producing a broad spectrum of cytokines, chemokines, growth factors, and interleukins [7]. Characterizing this heterogeneous environment should enable the identification of prognostic and predictive factors that can guide individualized treatment algorithms and limit or promote tumor progression. Immune TME features are also novel therapeutic targets such as checkpoint inhibitor immunotherapies, including cytotoxic T-lymphocyte-associated protein 4 (CTLA-4) or programmed death-1 (PD-1)/PD-L1 inhibitors [8,9].

One of the essential components of TME is the tumor-infiltrating lymphocytes (TILs) niche, which represents a selected lymphocyte population with enhanced specific immunological reactivity against tumor cells [10]. Numerous studies discuss the promising role of TILs as biomarkers of the immune response to the tumor. They present a better prognosis and higher survival rates in the TILs-rich melanoma [10,11]. According to the classical scoring system of Clark, based on hematoxylin and eosin-stained slides, TILs divide into three categories (brisk, non-brisk, or absent), and they should be reproducibly reported by the pathologist [10]. In contrast, some clinical trial results do not demonstrate an association of TILs with long-term outcomes and are questioning its significance [9,12,13]. One of the possible reasons for these controversial conclusions may be the methodology of TILs assessment. That is why several alternative scoring systems have been tested, for example, a four-tier TILs grading scheme, including the density and distribution of TILs infiltrate [14]. Moreover, experience from introducing Immunoscore in other solid tumors, for example, colon, lung, pancreatic, or hepatocellular cancer, supports the development of TILs evaluation tools based on digital image acquisition analysis [15,16].

TILs are a heterogeneous group of immunoinflammatory cells with different functional subsets [10]. The leucocyte subsets are visualized by detecting surface antigens by immunohistochemistry (IHC), immunofluorescence, or multiplex techniques supported by dedicated image analysis software [9,10]. CD8+ cytotoxic T-cells are present in primary melanoma and play a crucial role in the adaptive immune response by killing malignant cells, controlling tumor growth, and mediating the response to immunotherapy [9]. According to the latest studies, PD-1+ TILs represent the tumor-specific “exhausted” phenotype of CD4+ and CD8+ T cells [17,18]. Similarly, CD4+ T helper lymphocytes promote antitumor immune activation by cytolytic mechanisms or interferon-gamma (IFN-γ) secretion; other action methods include the crosstalk with B cells and stimulating effect on CD8+ cytotoxic lymphocytes [10]. One of the T-helper lineages is FOXP3+ regulatory cells, which were primarily classified as potentially immunosuppressive [19]. The results under the association of FOXP3+ TILs with prognosis in melanoma are conflicting [9,20,21]. The methodology of TILs subsets enumeration seems to be fundamental in achieving highly reproducible results. Standardization of pathological evaluation should be revised since inconsistent conclusions influence the potential value of TILs in melanoma prognosis.

However, the TILs value in cutaneous melanoma and its rare subtype in the Caucasian population, AM, remains unclear. Although many studies report cell infiltration as an important prognostic factor in primary melanoma patients [9,12,22], only a few investigate the AM subgroup [22,23,24]. Most data come from studies examining non-Caucasian patients, mostly Asians, where the incidence rate of AM is higher.

The aim of the study is to report a comprehensive analysis of TILs, FOXP3, CD4, CD8, PD1, and PD-L1, and their impact on survival in primary AM patients. Moreover, we try to indicate one preferred method of TILs evaluation. Herein, we report a comprehensive analysis of TILs, including their density, distribution, and immunophenotype in primary AM. We analyze all biomarkers in the context of patient survival rates and their prognostic value.

## 2. Materials and Methods

### 2.1. Study Population

We carried out a single-center retrospective cohort study based on predefined criteria. From 1998 to 2013, 70 patients diagnosed and treated in Maria Sklodowska-Curie National Research Institute of Oncology (MSNRI), Warsaw, Poland, with primary AM were consecutively enrolled in the study. Patients were selected and considered eligible for the study based on the following criteria: diagnosis of primary cutaneous melanoma after excisional biopsy with Breslow thickness ≥0.75 mm or presence of ulceration, feasibility for general anesthesia, availability of sentinel lymph node (SLN) biopsy, the availability of representative formalin-fixed, paraffin-embedded (FFPE) material from a treatment-naïve primary AM. The exclusion criteria were as follows: incomplete medical records, lack of histopathological material, metastatic disease at the moment of diagnosis, clinically palpable lymph nodes, and melanoma of unknown primary site. Patients with nonulcerated Breslow thickness <0.75 mm melanomas were excluded from analysis as not undergoing SLN biopsy and with very low risk of relapse. The clinical and histopathological data, including age, gender, location of the primary site, SLN biopsy status, pathological stage, presence of ulceration, Clark scale, mitotic index, histopathological subtype, vascular invasion, and nerve invasion, were analyzed. Assessment of tumor-infiltrating lymphocytes included Clark TILs scale, Melanoma Institute of Australia (MIA) grading TILs scale, MIA TILs distribution, and MIA TILs density, and assessment of CD4, CD8, FOXP3, and PD-1 PD-L1 expression on melanoma cells was performed.

### 2.2. Histopathological Evaluation

**Figure 1:**
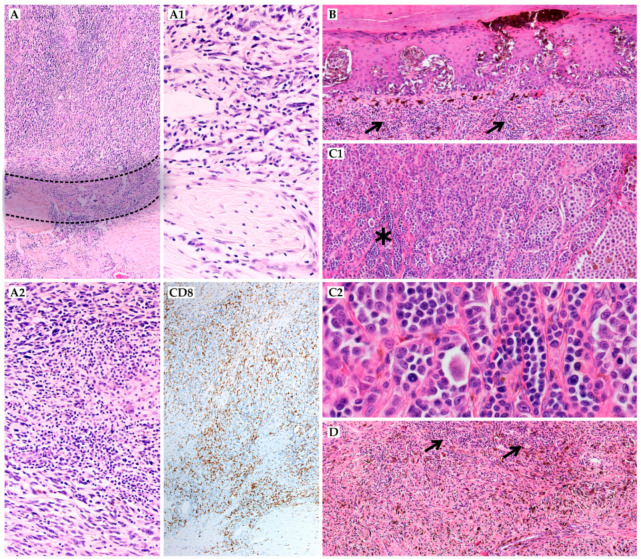
Histopathological evaluation of tumor-infiltrating lymphocytes (TILs); (**A**) brisk representative TILs according to Clark scale (HE, (haematoxylin–eosin), 20×), both intratumoral ((**A2**), HE, 200×) and peritumoral ((**A1**), HE, 200×) areas should be assessed; marked and diffuse **CD8**+ cells (20×); (**B**) in early stage melanomas (pT1) the brisk and dense TILs may cover up melanoma cells in vertical-growth phase (HE, 40×); (**C1**) (HE, 100×) and (**C2**) (HE, 400×)—some melanoma cells (see: asterisk) in low magnification may resemble TILs (see: arrows); (**D**) evaluation of TILs in highly melanocytic tumors may be challenging, in IHC the red chromogen or bleaching the slides may be helpful (HE, 20×).

All cases were reviewed and classified according to the 4th edition of the World Health Organization (WHO) diagnostic recommendations of skin tumors and the 8th edition of the AJCC [6]. Haematoxylin–eosin (HE) stained sections were reviewed by experienced pathologists (A.S.-C., M.C., and K.S.). The classical histopathological parameters included histopathological subtype, Breslow’s and Clark’s scales, presence of ulceration, number of mitotic figures per 1 mm^2^, vascular and perineural invasion, and evaluation of TILs. Quantification as absent, non-brisk, and brisk, as previously reported by Clark [25], and assessment according to the MIA grading of TILs [14] were implemented (Figure 1). The MIA evaluation was based on TILs density (mild, moderate, and marked) and distribution (focal, multifocal, and diffuse) in the tumor’s vertical-growth phase and in direct contact with melanoma cells as previously described. The four-tier MIA grading scale included the following: 0—TILs absent, 1—mild or moderate, focal or mild, multifocal TILs infiltrate, 2—marked focal or a moderate or marked, multifocal or mild, diffuse TILs infiltrate, 3—moderate or marked, diffuse TILs infiltrate. In case of discrepancy, a final consensus was achieved by reviewing the cases with a multiheaded microscope.

### 2.3. Immunohistochemistry

Immunohistochemistry assessment was performed for all 70 patients. The immunohistochemical analysis (IHC) was implemented to identify the T-cell immunoprofile of TILs in the microenvironment and evaluate the PD-L1 expression on melanoma cells. In all cases, positive and negative controls were included. IHC was performed on 4 μm thick sections with primary antibodies: anti-CD4 (RTU, pH 9.0, Dako/Agilent, Santa Clara, CA, USA), anti-CD8 (RTU, pH 9.0, Dako/Agilent), anti-FOXP3 (clone 236A/E7, 1:40, pH 9.0, Abcam, Cambridge, UK), anti-PD-1 (clone NAT105, 1:25, pH 9.0, Cell Marque, Rocklin, CA, USA), and anti-PD-L1 (clone 22C3, 1:50, pH 6.0, Dako/Agilent). The signals were retrieved with EnVision^TM^ FLEX (Dako/Agilent) in an automated Dako Autostainer, and sections were counterstained with hematoxylin. The positive controls included tonsil and PD-L1 IHC 22C3 pharmDx Control Cell Line Slide (Dako/Agilent); ready-to-use mouse negative control (a cocktail of mouse IgG1, IgG2a, IgG2b, IgG3, and IgM, Dako/Agilent) was provided for each primary antibody. All photographs were taken by an Olympus BX53 microscope and DP73 camera (Olympus, Tokyo, Japan).

**Figure 2:**
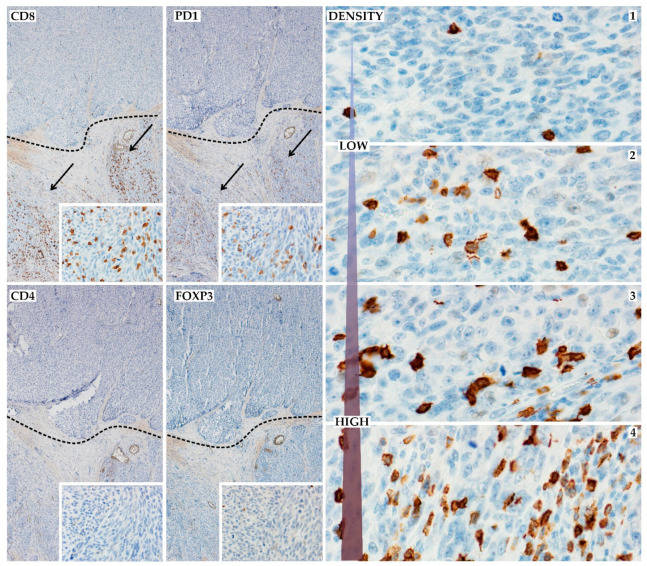
Immunohistochemical assessment of TILs; left panel—the TILs are localized only focally, the upper part of tumor lacks TILs, but the bottom part is characterized by marked TILs with immunophenotype: (**CD8**) and (**PD1**)-positive cells and without (**CD4**) and (**FOXP3**) expression (20× and 400× insert photos); right panel—TILs density in high magnification (anti-CD8 antibody, 400×), which is a simplified Immunoscore and division into the absent, low ((**1**) and (**2**)), or high ((**3**) and (**4**)) density of TILs.

Immunoscore for each T-cell compartment (CD4, CD8, FOXP3, and PD-1) was evaluated semiquantitatively, including density and localization (intratumorally and peritumoral—in the invasive margin of tumor) of positive antibody expression as previously described [26]. For further analysis, T-cells were categorized according to the simplified, practical scale: 0—lack of positive cells, 1—low (mild) positive cells content, and 2—high (moderate-to-severe) positive cells infiltration (Figure 2). Evaluation of PD-L1 was evaluated only on melanoma cells as previously described [27], and PD-L1-positive cases were defined as moderate or strong membrane staining in at least 5% of tumor cells.

### 2.4. Statistical Analysis

Pearson Chi-squared test or Fisher’s exact test (if frequencies ≤ 6) was used to analyze group proportions. Mann–Whitney *U* test was used to evaluate differences between continuous data. OS was calculated from the date of the first diagnosis to the last follow-up (censored) or death. The Kaplan-Meier method for estimating survival functions and the Cox proportional hazards model for estimating the effects of covariates on the hazard of the occurrence of death were used. All *p*-values < 0.05 were considered statistically significant. Data analysis was performed using the R software/environment (R Development Core Team. R: A Language and Environment for Statistical Computing; R Foundation for Statistical Computing: Vienna, Austria, 2009), version R 3.6.2, which is an open-source project that is distributed under the GNU General Public License [28].

## 3. Results

### 3.1. Clinicopathological Features

We analyzed 70 patients with primary AM at clinical stage I–II who underwent SLN biopsy between 1997 and 2014. All of the patients were Caucasian. The median follow-up was 84 months. Patients did not receive adjuvant systemic therapy with immune checkpoint inhibitors or targeted agents. At the time of the last follow-up, 41 patients had died. The mean age at the first diagnosis was 61.1 years (median: 64.5, 21–79 years). Our cohort included 47 females (67%) and 23 males (33%). Positive SLN biopsy was observed in 24 (34%) patients. Most lesions were located on foot (83%). The subungual location represented 36% of cases. According to the 8th edition of AJCC Melanoma of the Skin Staging, most patients were at the pathological stages II and III, 44% and 35%, respectively. Only 15 (21%) patients were in stage I.

The Breslow thickness of the primary lesion ranged from 0.1 to 25 mm (median 5 mm), and the majority of cases were classified as pT4 (60%). Most of the tumors were ulcerated (74%). The median mitotic count was 8.5 mitoses per 1 mm^2^. Vascular and nerve invasions were detected in 5 (7%) and 7 (10%) cases, respectively. The detailed clinical and histopathological characteristics of the patients are summarized in Table 1.

### 3.2. Histopathological and Immunohistochemical Features

TILs were evaluated in all 70 cases. Clark TILs grades were classified as absent, non-brisk, and brisk in 26%, 56%, and 18%, respectively. The four-tier scoring results on the Melanoma Institute of Australia (MIAs) TILs grades, including distribution and density profiles, showed: 0–26%, 1–33%, 2–24%, and 3–17%. The MIA TILs distribution was classified as focal—18%, multifocal—27%, and diffuse—29%; TILs density was described as absent, mild, moderate, and marked in 26%, 43%, 20%, and 11% of cases, respectively. The histopathological evaluation of TILs with the potential challenges was presented in Figure 1.

The high expression of CD8-, CD4-, FOXP3-, and PD-1-positive cells was detected in 48%, 31%, 26%, and 16% of cases, respectively (Figure 2, Supplementary Figure S1). Out of 70 patients, 41 (59%) presented expression of PD-L1 on melanoma cells (Supplementary Figure S2). The immunohistochemical characteristics of the patients are summarized in Table 2. Analyses of associations between immunohistochemical and clinicopathological features indicated TILs status and PD-1 status as strong significant predictors of SLN positivity (TILs Clark *p* = 0.02, TILs MIA grade *p* = 0.01, TILs MIA density *p* = 0.03, CD8 *p* < 0.01, CD4 *p* = 0.03, FOXP3 *p* < 0.01, and PD-1 *p* = 0.02). Several relationships were also discovered between immunohistochemical features and ulceration, AJCC Stage, subungual localization, and vascular invasion (Table 3).

### 3.3. Survival Analysis

**Figure 3:**
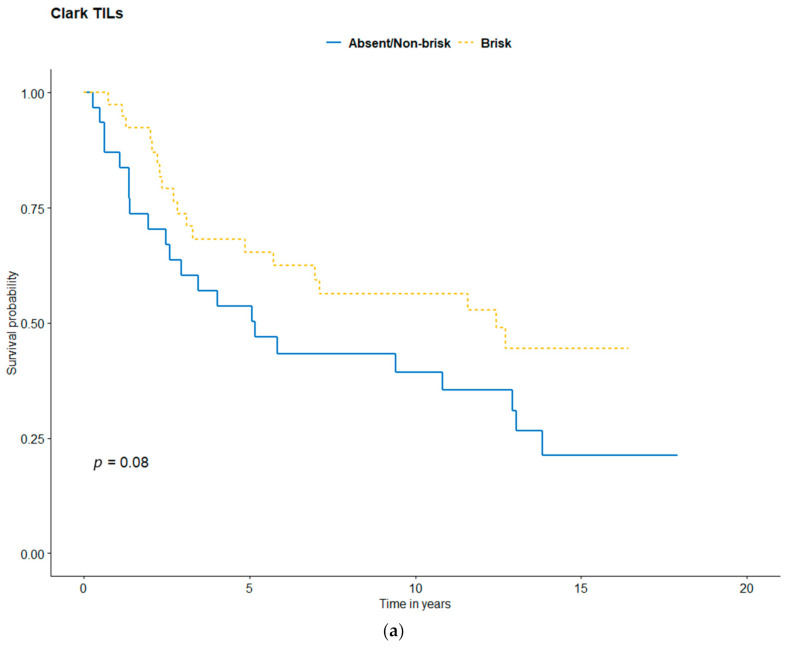

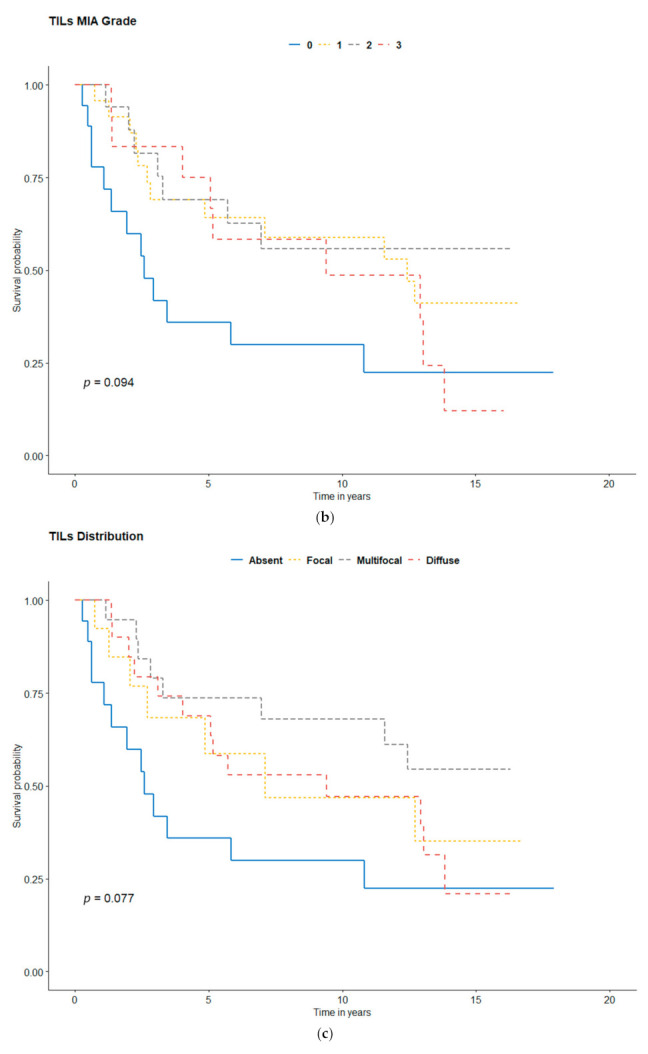

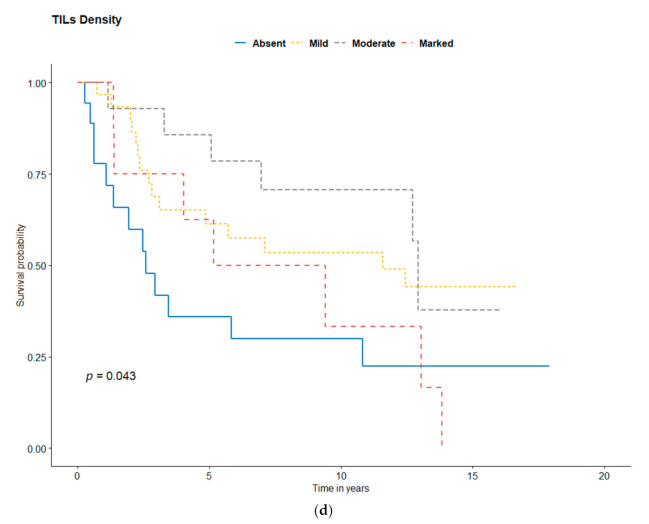
Kaplan-Meier survival curves for (**a**) TILs Clark: Absent/non-brisk vs. brisk, (**b**) TILs MIA grade, (**c**) TILs MIA distribution, and (**d**) TILs MIA density.

**Figure 4:**
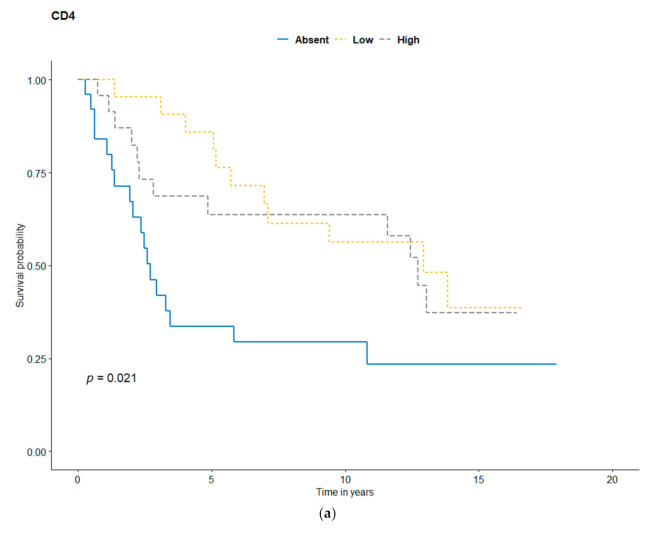

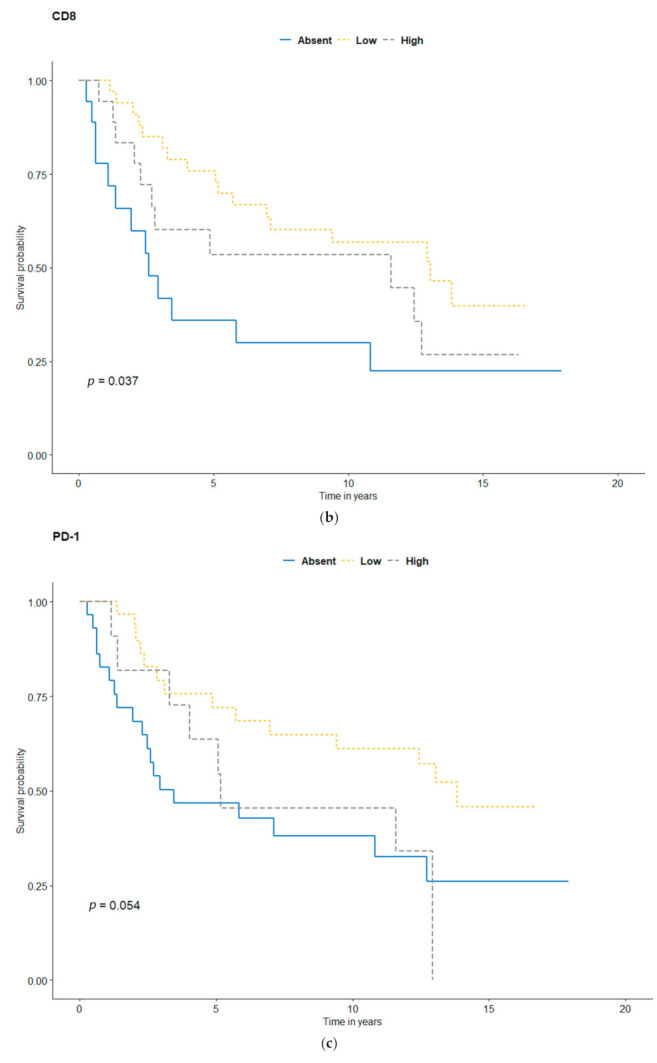

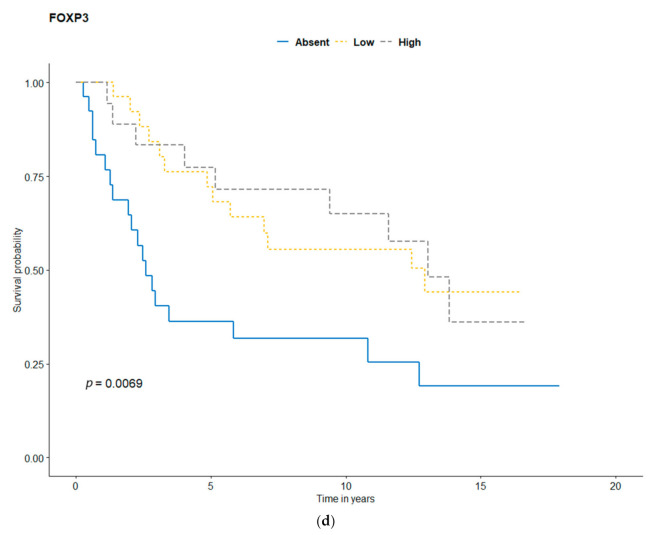
Kaplan-Meier survival curves for (**a**) CD4, (**b**), CD8, (**c**) PD-1, and (**d**) FOXP3.

**Figure 5:**
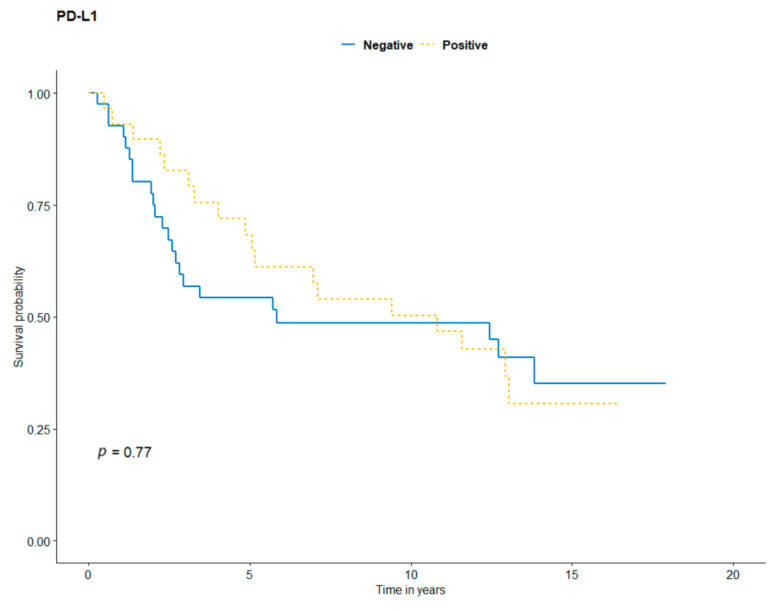
Kaplan-Meier survival curves for programmed death-ligand 1 (PD-L1) expression on acral melanoma and overall survival.

Kaplan-Meier analysis was performed to evaluate the patients’ survival curves, according to TILs, CD4, CD8, FOXP3, PD-1, and PD-L1 status (Figure 3, Figure 4 and Figure 5). In the Kaplan-Meier analysis, there were significant differences in OS depending on CD4+ (*p* = 0.021), CD8+ (*p* = 0.037), FOXP3+ T-cells (*p* = 0.007), and TILs density (*p* = 0.043). There was only a tendency for better outcomes for brisk Clark TILs (vs. absent/non-brisk, *p* = 0.08), TILs MIA grade (*p* = 0.094), TILs distribution (*p* = 0.077), and PD-1 (*p* = 0.054). The presence of PD-L1 expression was not correlated with survival (*p* = 0.770). Higher TILs counts correlated with better prognosis, with five-year OS rates for absent/non-brisk TILs and brisk TILs of 56% and 69%, respectively (Figure 3a). Additionally, CD4 and CD8 absence correlated with poorer survival outcomes than high CD4/CD8 expression did (Table 4, Figure 4a,b). The higher density of FOXP3-positive cells correlated with better survival (Figure 4d). There was no association between PD-L1 and survival.

To assess the impact of clinical, histopathological, and immunohistochemical features on OS prognosis, we performed univariate and multivariate Cox proportional hazards analyses. Univariate analysis showed that older age, pathological stage III, presence of ulceration, more advanced Breslow thickness, presence of nerve invasion, male sex, and higher mitotic index showed worse survival outcomes (*p* < 0.05) (Table 5). Univariate analysis of immunohistochemical features showed that FOXP3 and CD4 were the most associated markers with survival (Table 4). However, a significant correlation was also observed in CD8, PD-1, TILs MIA density, TILs MIA distribution, and TILs MIA grade.

**Figure 6:**
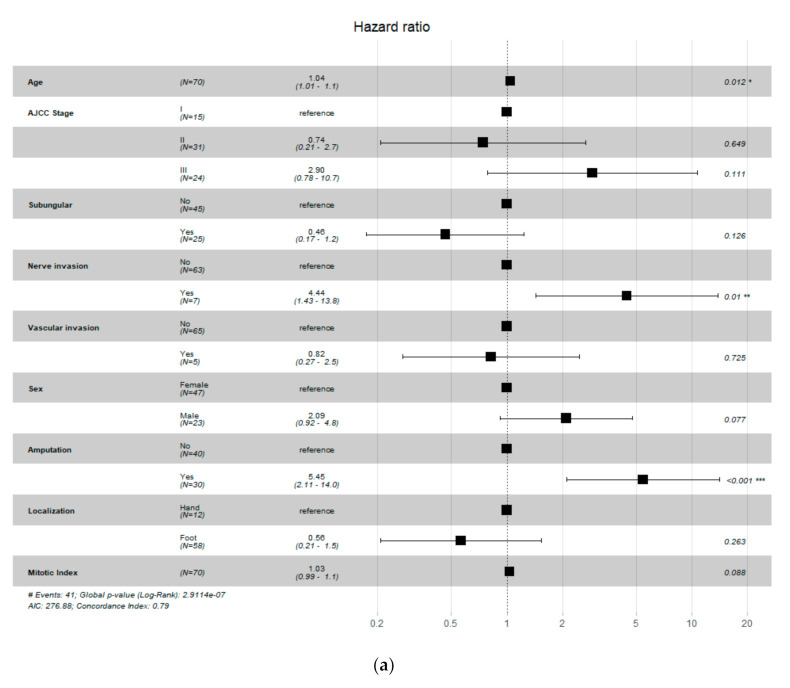

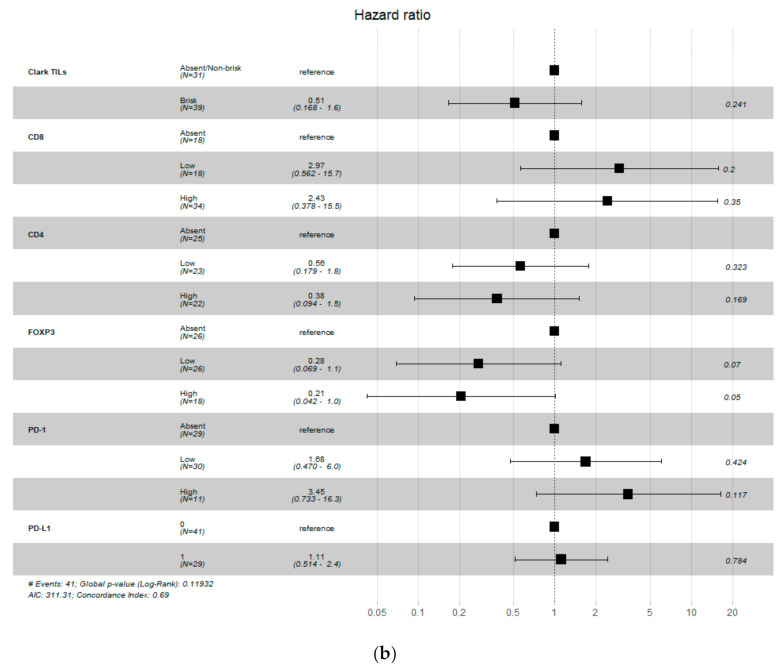
Multivariate analysis of (**a**) clinical and pathological features and (**b**) immunohistochemical features shown in Forest Plot. Significance code for *p*-value *** [0, 0.001] ** (0.001, 0.01] * (0.01, 0.05].

Multivariate analysis of clinical and pathological features showed that older age, nerve invasion, and amputation not performed were correlated with worse survival. Multivariate analysis of immunohistochemical features showed that only a higher density of FOXP3-positive cells was statistically significantly associated with better survival. Forest plots of multivariate analysis are shown in Figure 6.

## 4. Discussion

This study represented the largest single-institution study of AM in the Caucasian population with a long follow-up time. Our study showed that high TILs content in primary AM, classed mostly in MIA grade and density system, was associated with better outcomes (Figure 3; Figure 6). Moreover, SLN status was significantly correlated with immunohistochemical features (Table 3). Therefore, both can be potent markers of a favorable prognosis. Lee et al. divided melanomas into non-acral, non-nail acral, and nail unit melanomas. Analysis of separate subgroups showed prognostic significance between survival outcomes and density of TILs in both acral groups; however, there was no significant association of survival outcomes in non-acral patients [22]. A study of ALM (acral lentiginous melanoma) patients showed that favorable survival had only a trend to be associated with high TILs [23]. In the study of 90 Korean AM patients, the non-brisk TILs group was significantly correlated with a better prognosis than the absent TILs group. Interestingly there was only one patient (1.1%) described with brisk TILs [24]. Another analysis of 655 primary melanoma patients indicated that a higher TILs content was associated with a better prognosis. Authors found that in the AM subgroup, brisk TILs were less common than in the entire cohort. The five-year OS for a whole group for absent/non-brisk and brisk TILs was 80% and 88%, respectively [29]. Our patients achieved poorer survival outcomes; five-year OS for absent/non-brisk TILs was 56% and for brisk TILs was 69%. A meta-analysis of melanoma patients showed that the brisk infiltration of TILs was associated with improved OS [29]. An Australian study of 1138 all-sites melanoma patients showed TILs grade as an independent predictor of survival, with a 22% difference in five-year melanoma-specific survival between TILs grade 3 and TILs grade 0 patients, 100% and 78%, respectively [14]. On the contrary, a study involving 875 all-sites melanoma patients revealed that presence of TILs was not an independent predictive factor for survival, with five-year OS of 75% and 76% for TILs absent and TILs present, respectively [13]. We summarize the studies addressing the prognostic value of TILs on OS in AM/ALM patients in Table 6.

In our series of AM, we found that CD4, CD8, and FOXP3 on T-cells were correlated with better survival outcomes (Figure 4a,b,d). Moreover, a higher density of FOXP3-positive cells was an independent factor associated with better survival (Figure 6b). In Erdag et al.’s study of metastatic melanomas, CD8 was correlated with survival, but, unlike in our results, numbers of CD4+ and FOXP3+ cells were not associated with survival [9]. A meta-analysis of all-sites melanoma showed a favorable role of CD4+, CD8+, and FOXP3+ as prognostic factors [22]. Castaneda et al. studied 43 patients from Peru diagnosed with acral lentiginous melanoma (ALM), mainly located on hand and foot. Results showed that a higher density of CD4 TILs was associated with thinner Breslow thickness and longer survival rates (*p* = 0.005). Moreover, there was a tendency for more prolonged survival for higher CD8/CD3 [31]. Another study of 143 Peru patients with ALM also showed a trend for longer overall survival in high TILs cases (*p* = 0.09) [23]. We have found the tendency for better survival outcomes for the presence of PD-1-positive T-cells (Figure 4c). Similar to our findings, Lee et al. reported on PD-1 expression AM patients; however, there was no significant association in the non-acral group [22]. Erlag et al. also reported that PD-1 was correlated with survival, but the analysis was performed in metastatic melanoma patients [9].

Although PD-L1 expression provided significant value to predict response to immunotherapy and predicted prognosis in several cancer types [32,33,34,35,36], its significance in acral melanoma remains unclear. In our analysis, OS in AM was independent of PD-L1 expression on tumor cells (Figure 5). However, we found statistically significant correlations between PD-L1 status and TILs characteristics (Supplementary Table S1). Evaluation of PD-L1 should also be investigated in the future for expression on melanoma cells and stromal cells; this will require more extensive immunohistochemical techniques, including multiplex IHC. Ren et al. evaluated 78 Chinese patients with primary acral melanoma. The study showed that PD-L1 expression on TILs was an independent predictor of poor prognosis. However, no effect on survival was demonstrated in the case of PD-L1 expression on tumor cells [30]. Our findings indicated no correlation between PD-L1 expression on melanoma cells and survival rates. Although our rates of presence of PD-L1 expression were higher than in a Ren et al. study, 59% vs. 37.2%, respectively [30]. Moreover, we indicated that PD-L1 expression has no impact on clinical or pathological features (Table 3).

The critical issue is the heterogeneity of compared melanoma subgroups. We showed that in AM, TILs assessment techniques could be applied differently; some authors are basing only on HE assessment or implement immunohistochemical profiling with different cut-offs systems (Table 6). Moreover, the predictive value of TILs can be limited due to incoherent categorization of tumor stages (AJCC and SLN status) and basic pathological features (i.e., Breslow level and ulceration) [23,24,30,31]. Several results cannot be compared because of inconsistent patient inclusion criteria and unclear scoring methods. Lately, multiplex immunohistochemical studies and digital imaging analysis are rapidly developing tools for TME and TILs evaluation [37,38]. We believe that the comparative results of TILs assessment in “classical” HE and IHC and “new” techniques may be beneficial for pathological revision and unification of methodology. Strengthening the conclusions on TILs prognostic value and more in-depth insight into differences between patients should be guided by harmonized recommendations.

In our study, the association between immunohistochemical and clinicopathological features demonstrated that TILs are significant biomarkers in SLN status prediction (Table 3). These results are in line with previous studies of all sites of melanomas. Taylor et al. studied 875 patients diagnosed with melanoma and assessed factors that predict SLN positivity and survival. The metastatic SLN was observed in 17.6% of patients. Multivariate analysis showed that Breslow thickness, ulceration, and absent TILs were independently predictive of positive SLN. The probability of a positive SLN was 3.9% in the group with brisk TILs infiltrate and 26.2% in the group with absent TILs [13]. Another study of 1138 melanoma patients showed TILs grade as an independent predictor of SLN status. SLN positivity was observed in 27.8% of patients with an absence of TILs compared to 5.6% of patients with TILs grade 3 [14]. Interestingly, some studies investigated sex-specific prognostic implications of TILs, with miscellaneous relevance. The results of 1367 localized primary melanoma patients showed that SLN positivity was associated with TILs in the males (*p* < 0.001), but there was no association among women (*p* = 0.49) [39]. Another study presented 16.9% positive SLN among 851 melanoma patients. In multivariate analysis, TILs status was an independent predictor of SLN. The analysis stratified by sex pointed out that the statistically significant prediction was observed only for women (*p* = 0.037) but not for men (*p* = 0.172) [40]. In our study, we have confirmed a significant association between the SLN status and TILs, assessed both in HE staining (Clark and MIA grade scores) and immunohistochemically (Table 3). High levels of TILs were seen among patients with negative SLN, while the immunoprofile seemed less critical. 

The points of strength of our study are a unique and large group of acral melanoma cases, homogeneous treatment and follow-up in one institution, and comprehensive TILs evaluation according to widely applied scoring systems. The limitations include the unavailability of digital analysis implementation and the scantiness of publications that could be compared. There were also limitations related to the statistical power of multivariate analyses, which in our group were only 40 events.

## 5. Conclusions

In conclusion, we show that TILs in AM play an essential role in survival prediction. Mostly TILs MIA grade and density system are associated with better outcomes. Moreover, CD4, CD8, and FOXP3 on T-cells are correlated with better survival outcomes. However, we cannot indicate one preferred method of TILs evaluation. Both traditional HE-based evaluation and immunophenotyping are related to the prognosis of patients. Our findings show no correlation between PD-L1 expression on melanoma cells and clinicopathological features or survival rates. We consider that the consensus of scoring methods is an important issue that is needed to achieve more comparable results in TILs evaluation.

## Figures and Tables

**Table 1:** Baseline clinical and histopathological features of melanoma cases.

Clinical Characteristic	
Features	Number (%)
Age (median = 64.5; range = 21–79)	
Breslow thickness (median = 5; range = 0.1–25)	
Mitotic index (median = 8.5 mitoses per 1 mm^2^; range = 1–41)	
Gender	
Female	47 (67)
Male	23 (33)
Localization	
Hand	12 (17)
Foot	58 (83)
Subungual localization	
No	45 (64)
Yes	25 (36)
Amputation	
No	40 (57)
Yes	30 (43)
SLN biopsy	
Negative	46 (65)
Positive	24 (35)
AJCC Pathologic Stage	
I	15 (21)
II	31 (44)
III	24 (35)
Ulceration	
No	18 (26)
Yes	52 (74)
pT	
pT1	
a	7 (10)
b	2 (3)
pT2	
a	7 (10)
b	2 (3)
pT3	
a	3 (4)
b	7 (10)
pT4	
a	1 (1)
b	41 (59)
Clark scale	
1	4 (6)
2	5 (7)
3	6 (8)
4	28 (40)
5	27 (39)
Vascular Invasion	
No	65 (93)
Yes	5 (7)
Nerve Invasion	
No	63 (90)
Yes	7 (10)

SLN—sentinel lymph node, AJCC—American Joint Committee on Cancer.

**Table 2:** Baseline immunohistochemical features of melanoma cases.

Tumor-Infiltrating Lymphocytes Characteristics	
Feature	Number (%)
TILs Clark	
Absent	18 (26)
Non-brisk	39 (56)
Brisk	13 (18)
TILs MIA Grade	
0	18 (26)
1	23 (33)
2	17 (24)
3	12 (17)
TILs MIA Density	
Absent	18 (26)
Mild	30 (43)
Moderate	14 (20)
Marked	8 (11)
TILs MIA Distribution	
Absent	18 (26)
Focal	13 (18)
Multifocal	19 (27)
Diffuse	20 (29)
CD4	
Absent	25 (36)
Low	23 (33)
High	22 (31)
CD8	
Absent	18 (26)
Low	18 (26)
High	34 (48)
FOXP3	
Absent	26 (37)
Low	26 (37)
High	18 (26)
PD-1	
Absent	29 (41)
Low	30 (43)
High	11 (16)
PD-L1 on melanoma cells	
Positive	41 (59)
Negative	29 (41)

TILs—tumor-infiltrating lymphocytes, MIA—Melanoma Institute of Australia, programmed death-1 (PD-1), programmed death-ligand 1 (PD-L1).

**Table 3:** Association between immunohistochemical and clinicopathological features. Statistical significance (*p* ≤ 0.05) is marked with *.

	TILs Clark	TILs MIA Grade	TILs MIA Density	TILs MIA Distribution	CD8	CD4	FOXP3	PD-1	PD-L1
Ulceration	0.20	0.36	0.34	0.44	0.22	0.08	0.05 *	0.50	1.00
AJCC Stage	0.20	0.29	0.11	0.45	0.03 *	<0.01 *	0.62	0.33	0.21
Gender	0.98	0.40	0.77	0.83	0.77	0.46	0.33	0.45	0.62
Localization	0.22	0.14	0.38	0.55	0.08	0.12	0.15	0.83	0.34
SLN Status	0.02 *	0.01 *	0.03 *	0.08	<0.01 *	0.03 *	<0.01 *	0.02 *	0.73
Subungual	0.27	0.10	0.40	0.23	0.01 *	0.18	0.33	0.74	0.94
Nerve invasion	0.55	0.61	0.70	0.50	0.30	0.30	0.30	0.96	0.63
Vascular Invasion	0.95	0.97	0.93	0.34	0.88	0.92	0.50	0.02 *	0.18

TILs—tumor-infiltrating lymphocytes, MIA—Melanoma Institute of Australia, AJCC—American Joint Committee on Cancer, and SLN—sentinel lymph node.

**Table 4:** Univariate analysis of immunohistochemical features. Statistical significance (*p* ≤ 0.05) is marked with *.

Feature	Value	Hazard Ratio	CI.95	*p*-Value
Clark TILs	Absent/Non-brisk	-		
	Brisk	0.58	0.31–1.10	0.084
TILs MIA Grade	0	-		
	1	0.45	020–0.99	0.046 *
	2	0.37	0.15–0.92	0.033 *
	3	0.62	0.26–1.45	0.271
TILs MIA Density	Absent	-		
	Mild	0.45	0.21–0.95	0.036 *
	Moderate	0.32	0.12–0.84	0.021 *
	Marked	0.83	0.33–2.08	0.687
TILs MIA Distribution	Absent	-		
	Focal	0.52	0.21–1.31	0.167
	Multifocal	0.33	0.14–0.79	0.013 *
	Diffuse	0.58	0.27–1.25	0.161
CD8	Absent	-		
	Low	0.62	0.28–1.40	0.251
	High	0.4	0.19–0.82	0.013 *
CD4	Absent	-		
	Low	0.48	0.23–1.00	0.048 *
	High	0.39	0.18–0.82	0.013 *
FOXP3	Absent	-		
	Low	0.39	0.19–0.79	0.009 *
	High	0.38	0.17–0.83	0.016 *
PD-1	Absent	-		
	Low	0.44	0.22–0.89	0.022 *
	High	0.89	0.39–2.04	0.785
PD-L1	Absent	-		
	Positive	0.91	0.49–1.69	0.771

CI.95—95% confidence interval, TILs—tumor-infiltrating lymphocytes, MIA—Melanoma Institute of Australia.

**Table 5:** Univariate analysis of clinical and histopathological features. Statistical significance (*p* ≤ 0.05) marked with *.

Feature	Value	Hazard Radio	Cl.95	*p*-Value
Age	Mean (SD)	1.06	1.02–1.09	0.001 *
AJCC Stage	I	-		
	II	2.76	0.91–8.34	0.073
	III	7.98	2.70–23.56	<0.001 *
Ulceration	no	-		
	yes	5.52	1.96–15.59	0.001 *
Breslow thickness	Mean (SD)	1.13	1.06–1.19	<0.001 *
Subungular	no	-		
	yes	1.64	0.87–3.09	0.125
Nerve invasion	no	-		
	yes	3.87	1.60–9.36	0.003 *
Vascular invasion	no	-		
	yes	1.50	0.54–4.30	0.43
Sex	female			
	male	2.29	1.22–4.30	0.010 *
Amputation	no			
	yes	1.85	1.00–3.44	0.051
Localization	hand			
	foot	0.48	0.23–1.02	0.058
Mitotic Index	Mean (SD)	1.05	1.02–1.8	<0.001 *

AJCC—American Joint Committee on Cancer, CI.95—95% confidence interval.

**Table 6:** Summary of studies addressing TILs in acral melanoma. Statistical significance (*p* ≤ 0.05) is marked with *. The acral lentiginous melanoma (ALM) patient populations are marked with **.

Number of Cases	Clinical Characteristics					Histopathological Characteristics			TILs Characteristics			Prognostic Value of TILs on OS	Reference Number/Year
	Age (Median)	Population	F:M	Localization	AJCC Stage	Breslow (mm, Median)	Ulceration Present	Positive SLN	TILs Evaluation	Methods	IHC Profile		
148	66	Peru **	1.21	NA	0–III	6.0	69%	54.2%	TILs grade	IHC	CD3, CD8, p16	p16 *p* = 0.001 *	Castaneda et al. [23]/2019
78	61.5	Chinese	0.63	Hand (6%) Foot (94%)	I–IV	NA	45%	35%	TILs distribution Extent of TILs infiltration (<5% vs. ≥5%)		PDL-1	PD-L1 expression in TILs *p* = 0.008 *	Ren et al. [30]/2018
90	61.3	Korean	1.09	NA	NA	NA	NA	NA	TILs absent, non-brisk, brisk	HE		TILs absent *p* = 0.037 *	Lee et al. [24]/2013
43	66.7	Peru **	0.45	NA	I–III	5.0	74%	44.2%	TILs density TILs distribution TILs grade		CD3, CD4, CD8, CD20, CD68, CD163	CD4 *p* = 0.005 *	Castaneda et al. [31]/2017
70	64.5	Caucasian	2.04	Hand (17%) Foot (83%)	I–III	5.0	74%	34%	TILs Clark; TILs MIA Grade; TILs Absent vs. Low/High	HE IHC	CD4, CD8, FOXP3, PD-1,	TILs Clark *p* = 0.08; TILs MIA Grade *p* = 0.094; TILs MIA Distribution *p* = 0.077; TILs MIA Density *p* = 0.043 *; CD8 *p* = 0.03 *; CD4 *p* = 0.02 *; FOXP3 *p* = 0.006 *; PD-1 *p* = 0.05 *	Presented study/2021

AJCC—American Joint Committee on Cancer, ALM—acral lentiginous melanoma, TIL—tumor-infiltrating lymphocyte, SLN—sentinel lymph node, F—female, M—male, HR—hazard ratio, IHC—immunohistochemistry, MIA—Melanoma Institute of Australia, OS—overall survival, HE—Haematoxylin–eosin, and NA—not available.

## Data Availability

Data available on request due to restrictions, privacy or ethical.

## Supplementary Materials

The following are available online at https://www.mdpi.com/article/10.3390/jcm10071452/s1, Figure S1: The high magnification images of TILs immunoprofile, Figure S2: PD-L1 immunohistochemical expression pattern, Table S1: PD-L1 expression status in correlation with TILs characteristics.
