# Supplementary material for: Clinical Significance of Tumor Microenvironment in Acral Melanoma: A Large Single-Institution Study of Caucasians
Source: J Clin Med. 2021 Apr 1;10(7):1452. doi: 10.3390/jcm10071452 (PMC8036823; doi:10.3390/jcm10071452)
Supplement: Supplementary file 1 [file jcm-10-01452-s001.pdf]

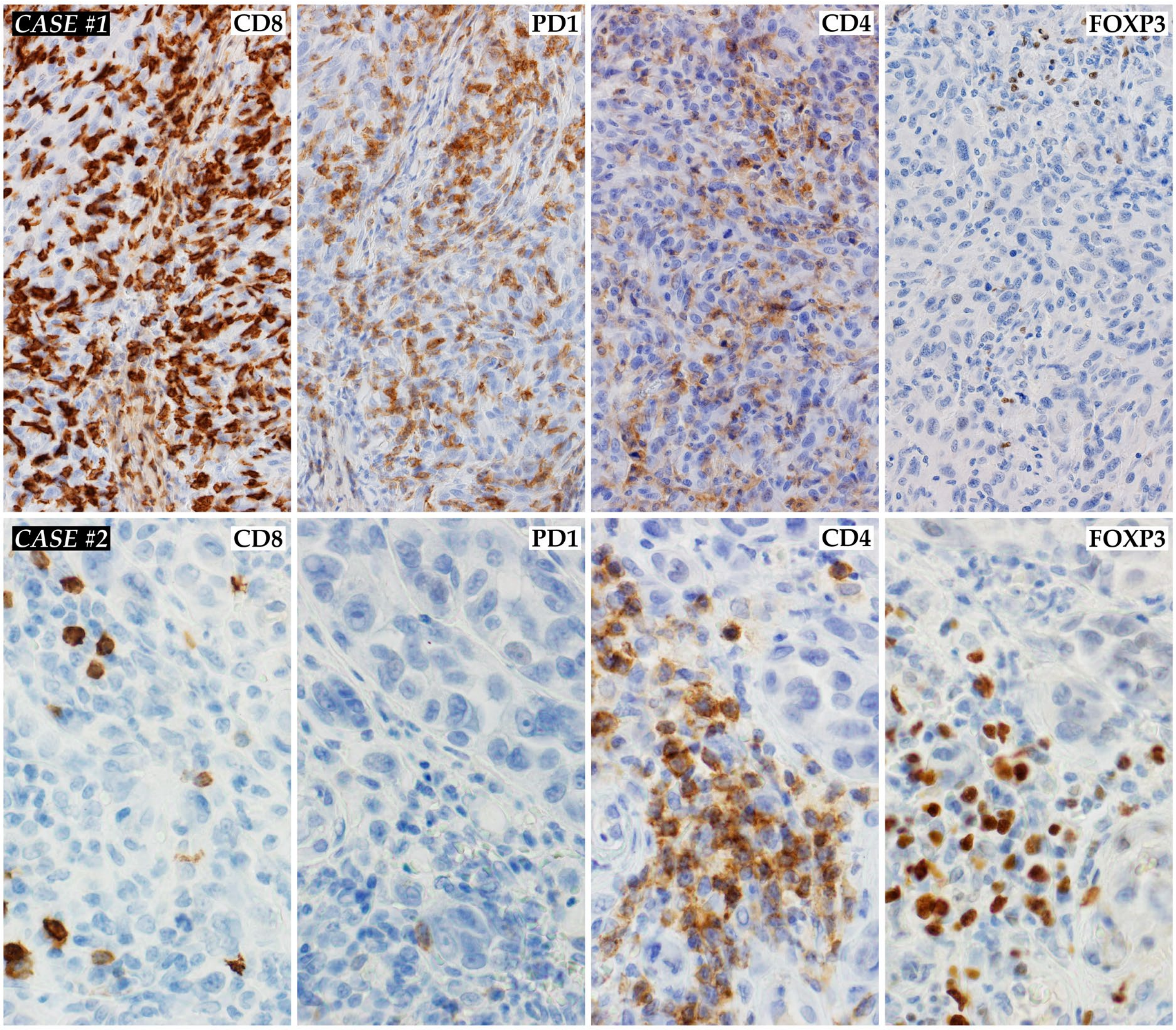

Supplementary Figure 1. The high magnification images of TILs immunoprofile; Case #1 was characterized with increased infiltration of CD8(+) and PD1(+) cells, lower CD4(+) cells, and sparse population of FOXP3(+) cells (200x); Case #2 was accompanied with CD4(+) and FOXP(+) T-cells infiltration with the low density of CD8(+) and PD1(+) cells (400x).

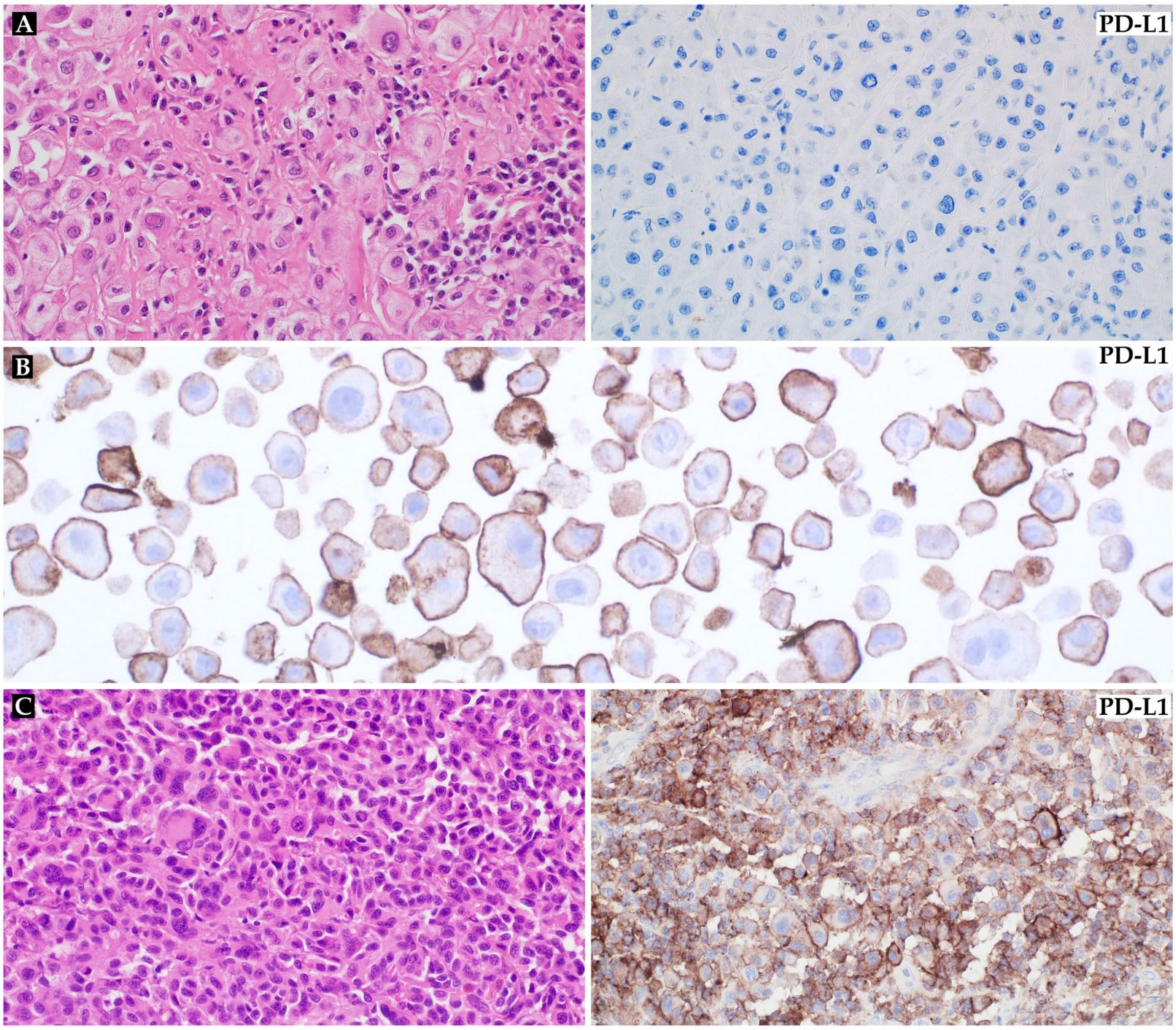

Supplementary Figure 2. PD-L1 immunohistochemical expression pattern: the negative (A, 200x) and positive (C, 400x) case with positive control „in-between" (B, 200x).

Supplementary Table 1. PD-L1 expression status in correlation with TILs characteristics. Statistical significance ($p \leq 0.05$) was bolded and marked with *.

	TILs Clark	TILs MIA Grade	TILs MIA Density	TILs MIA Distribution	CD8	CD4	FOXP3	PD-1
PD-L1	**0.01***	**0.02***	**0.03***	**0.05***	**0.01***	**0.01***	**0.01***	**0.01***

TILs - tumor-infiltrating lymphocytes, MIA – Melanoma Institute of Australia
